# The association between malaria prevalence and COVID-19 mortality

**DOI:** 10.1186/s12879-021-06701-8

**Published:** 2021-09-19

**Authors:** Michael U. Anyanwu

**Affiliations:** Department of Community Health, Mbarara University of Science and Technology, P.O Box 1410, Mbarara, Uganda

**Keywords:** Covid-19, Mortality, Malaria, Prevalence, Ecological study

## Abstract

**Background:**

The number of persons infected with COVID-19 continues to increase with deaths reported daily across the globe. High income countries such as the US, the UK, Italy and Belgium have reported high COVID-19 related deaths but low-and-middle-income countries have recorded fewer deaths despite having poor healthcare system. This study aimed to investigate the association between malaria prevalence and COVID-19 mortality.

**Methods:**

An ecological study was conducted with data from 195 countries. Spearman’s correlation was used to test the association between the population variables and COVID-19 mortality. Generalized linear model with Poisson distribution was used to determine the significant predictors of COVID-19 mortality.

**Results:**

There was a significant positive correlation between median age, life expectancy, 65+ mortality and COVID-19 mortality while malaria prevalence, sex ratio and cardiovascular mortality were negatively correlated with COVID-19 mortality. Malaria prevalence, life expectancy and mortality rate were significant on multivariate regression analysis.

**Conclusion:**

The results of this study support the hypotheses that there are reduced COVID-19 deaths in malaria endemic countries, although the results need to be proved further by clinical trials.

## Background

Coronavirus disease 2019 (COVID-19) is an infectious disease which is caused by a new coronavirus known as SARS-CoV-2 (Severe Acute Respiratory Syndrome Coronavirus 2) [1]. The outbreak began in Hubei province of China in December 2019, and it was announced as a Public Health Emergency of International Concern on January 2020, and later on March 2020, proclaimed a pandemic by the World Health Organization (WHO) after more than 4291 deaths were recorded in many countries [2]. COVID-19 is transmitted through close contact with infected droplets from sneezing, coughing, talking or breathing [3]. The signs and symptoms includes fever or chills, cough, shortness of breath or difficulty in breathing, fatigue, headaches, new loss of taste or smell, sore throat among others [3]. Hand hygiene, social distancing, isolation and use of face masks have been recommended by WHO as well as other preventive measures such as local and international travel restrictions and lockdowns in order to stop the spread of COVID-19 [4].

As at April 20, 2021, there were about 3,021,397 COVID-19 related deaths reported globally [5]. In various countries, differences in COVID-19 mortality has been observed with most Western countries having high mortality per million population [6]. For example, the mortality per million population in the USA is 1697, the United Kingdom (1874), France (1538) and Belgium (2060). However, in developing nations, low COVID-19 mortality have been reported with Nigeria having a mortality per million population of 10, Kenya (46), India (130) and Venezuela (67). This is in contrast to what was speculated in low-and-middle income countries where the morbidity and mortality of COVID-19 was expected to be high compared to Western countries [7]. Epidemiological studies have identified several factors associated with COVID-19 mortality. A study reported that the mean age, life expectancy and other population characteristics in African countries account for the low COVID-19 mortality [8]. Another ecological study of COVID-19 mortality across 173 countries showed that median age of the population and life expectancy were associated with COVID-19 mortality [6]. Pre-existing conditions have also been found to be associated with COVID-19 deaths. The Chinese Centre for Disease Control and Prevention reported in a study that cardiovascular disease, diabetes, hypertension and cancers were associated with an increased risk of COVID-19 death [9].

There has been widespread speculation that hydroxychloroquine a drug used to treat malaria can be used to prevent and treat COVID-19. Although some studies reported that it may inhibit COVID-19 virus, other studies showed that it has no positive effect on COVID-19. Scientists have argued that its use in some countries is responsible for the low COVID-19 deaths in those countries, and studies conducted during the early period of the pandemic revealed that there is reduced spread of COVID-19 in malaria endemic countries [10]. Following this assumption, the hypotheses that immune response against malaria in malaria endemic countries may have protective effects against COVID-19 was proposed.

## Materials and methods

### Study design

This is an ecological study which compared population variables of 195 countries.

### Data resources

All data were obtained from open resources. Information on COVID-19 was obtained from the WHO Coronavirus Disease Situation Dashboard [5]. Malaria data was obtained from the World Malaria Report 2020 [11]. All data regarding the population variables were obtained from the United Nations database [12], and data regarding cardiovascular mortality rate was culled from ‘Our World in Data’ [13].

### Dependent variable

The dependent variable evaluated in this study was COVID-19 mortality (total COVID-19 related deaths per million population) in each county obtained from the WHO Coronavirus Disease Situation Dashboard on 20th April, 2021 [5].

### Independent variable

Malaria prevalence was converted to total malaria cases per million population in each country. The data on malaria morbidity was culled from the World Malaria Report 2020 [11].

### Covariates

The covariates assessed in this study were median age, life expectancy at birth, mortality rate per 1000 population, 65+ mortality rate, sex ratio and cardiovascular mortality per 100,000 obtained from the United Nations database and ‘Our World in Data’ [12, 13].

### Statistical analysis

STATA Version 12 was used for all statistical analysis. Spearman’s correlation was used to test the association between COVID-19 mortality and the population variables. Generalized linear model (GLM) with Poisson distribution and log link was used to determine the significant predictors of COVID-19 mortality rate (deaths per million population). The Generalized linear model was used by reason of the zero values and non-parametric distribution of the dependent variable. The level of significance was set at p < 0.05 and 95% confidence interval.

### Ethics approval

Approval from institutional review board was not sought for this study due to the use of publicly available data from open resources.

## Results

A total of 195 countries that had data on COVID-19 deaths, malaria cases and population variables were included in the study. Tables 1 and 2 show the countries with the highest and lowest COVID-19 deaths per million populations and their population characteristics. Czechia (2668), Hungary (2598) Bosnia and Herzegovina (2398) and Montenegro (2283) have the highest COVID-19 deaths per million population while Samoa (0), Turkmenistan (0) and Vanuatu (0) are among the countries with no COVID-19 mortality. Among malaria endemic countries, Benin (391065), Burkina Faso (371174), Liberia (353937) and Rwanda (352794) have the highest malaria cases per million population.

**Table 1:** Population characteristics of countries with highest COVID-19 mortality per million population

Country	Covid deaths	Malaria prevalence	Median age	Sex ratio	Life expectancy	Mortality rate	65+ mortality	CVD mortality
Czechia	2668	0	43	97	79	11	82	227
Hungary	2598	0	43	91	77	13	76	278
Bosnia and Herzegovina	2398	0	43	96	77	11	78	329
Montenegro	2283	0	39	98	77	11	79	387
Bulgaria	2185	0	45	94	75	15	79	424
North Macedonia	2132	0	39	100	76	10	77	322
Slovenia	2129	0	45	99	81	10	83	153
Belgium	2060	0	42	98	81	10	84	114
Slovakia	2047	0	41	95	77	10	75	287
Italy	1960	0	47	95	83	11	89	113
UK	1874	0	41	98	81	9	84	122
Brazil	1756	834	34	97	76	6	56	177
Peru	1735	1378	31	99	76	6	60	85
USA	1697	0	38	98	79	9	74	151
Mexico	1646	5	29	96	75	6	57	152

**Table 2:** Population characteristics of countries with lowest COVID-19 mortality per million population

Country	Covid deaths	Malaria prevalence	Median age	Sex ratio	Life expectancy	Mortality rate	65+ mortality	CVD mortality
Micronesia	0	0	24	103	68	7	47	454
Tonga	0	0	22	100	71	7	61	227
Kiribati	0	0	23	97	68	6	31	434
North Korea	0	186	35	96	72	9	65	321
Laos	0	1434	24	101	67	7	41	368
Turkmenistan	0	0	27	97	67	7	40	536
Samoa	0	0	22	107	73	5	60	348
Vanuatu	0	3371	21	103	70	5	54	546
Solomon Island	0	236,540	20	104	73	4	44	459
Viet Nam	0.4	99	33	100	75	6	56	245
Tanzania	0.4	106,600	18	100	65	7	25	217
Burundi	0.5	283,023	17	99	61	8	18	293
Bhutan	1.3	3	28	113	71	6	44	217
Thailand	1.5	51	28	95	77	8	62	109
Timor-Leste	1.5	0	21	102	69	6	46	335

COVID-19 mortality per million population has a significant negative correlation with malaria prevalence (r = − 0.499, p < 0.001), cardiovascular deaths per 100,000 (r = − 0.305, p < 0.001) and sex ratio (− 0.323, p < 0.001). There was a significant positive correlation between COVID-19 mortality and median age (r = 0.630, p < 0.001), life expectancy (r = 0.580, p < 0.001) and 65+ mortality rate (r = 0.605, p < 0.001). Details are shown in Table 3.

**Table 3:** Spearman’s correlation of COVID-19 mortality, malaria prevalence and population variables

Variables		Covid cases	Covid deaths	Malaria prevalence	Median age	Sex ratio	LE	Mortality rate	65+ mortality	CVD mortality
Covid cases	R	1								
	p									
Covid deaths	R	0.934	1							
	p	0.000								
Malaria prevalence	R	− 0.577	− 0.499	1						
	p	0.000	0.000							
Median age	R	0.682	0.630	− 0.775	1					
	p	0.000	0.000	0.000						
Sex ratio	R	− 0.242	− 0.323	0.144	− 0.317	1				
	p	0.001	0.000	0.051	0.000					
Life expectancy	R	0.658	0.580	− 0.744	0.860	− 0.118	1			
	p	0.000	0.000	0.000	0.000	0.109				
Mortality rate	R	0.151	0.203	− 0.008	0.283	− 0.494	− 0.080	1		
	p	0.041	0.006	0.908	0.000	0.000	0.281			
65+ mortality	R	0.659	0.605	− 0.790	0.925	− 0.274	0.913	0.192	1	
	p	0.000	0.000	0.000	0.000	0.000	0.000	0.009		
CVD mortality	R	− 0.350	− 0.305	0.192	− 0.385	0.063	− 0.586	0.104	− 0.432	1
	p	0.000	0.000	0.009	0.000	0.398	0.000	0.161	0.000	

Malaria prevalence and all the covariates were significant in bivariate analysis, however, multivariate analysis showed that malaria prevalence (p < 0.001; CI 0.99–0.99), mortality rate (p = 0.001; CI 1.10–1.46) and life expectancy (p = 0.040; CI 1.01–1.25) were significantly associated with COVID-19 mortality. Details of the regression analysis are shown in Table 4.

**Table 4:** Regression analysis of predictors of COVID-19 mortality

Variable	Crude			Adjusted		
	IRR	p value	95% CI	IRR	p value	95% CI
Median age	1.09	< 0.001	1.07–1.11	0.97	0.376	0.91–1.03
Sex ratio	0.98	0.041	0.95–0.99	0.99	0.892	0.99–1.01
Life expectancy	1.12	< 0.001	1.09–1.14	1.12	0.040	1.01–1.25*
Mortality rate	1.16	< 0.001	1.11–1.22	1.27	0.001	1.10–1.46**
65+ mortality	1.04	< 0.001	1.03–1.05	0.99	0.637	0.96–1.02
CVD mortality	0.99	0.002	0.99–0.99	0.99	0.198	0.99–1.00
Malaria prevalence	0.99	< 0.001	0.99–0.99	0.99	< 0.001	0.99–0.99***

*IRR* incidence rate ratio

^*^p < 0.05; **p < 0.01; ***p < 0.001

## Discussion

This ecological study aimed to determine the association between COVID-19 mortality and malaria prevalence. After adjusting for the population dynamics, malaria prevalence was significantly associated with COVID-19 mortality. The results of this study is consistent with previous studies which revealed that malaria endemic countries have reduced COVID-19 morbidity and mortality [10, 14, 15].

The association between COVID-19 and malaria prevalence could be as a result of immune response against malaria in malaria endemic countries, and the extensive use of chloroquine drugs and its derivatives. Several studies have revealed that it is plausible to have cross immunity, where one pathogen can provide immunity against another pathogen [16, 17]. For example, prior exposure to *Plasmodium* has been shown to have protective effects against Chikungunya [18]. Studies have discovered that SARS-CoV-2 uses the angiotensin-converting enzyme 2 (ACE2) receptor to invade the host cells [19]. Similarly, studies have revealed that ACE1 and ACE2 polymorphisms protects the host from susceptibility to malaria [20, 21]. Furthermore, studies have indicated that interferons generated by lymphocytes as an immune response to malaria have in vitro and in vivo efficacy against the coronavirus responsible for COVID-19 [22, 23]. The therapeutic role of chloroquine and its derivatives in COVID-19 infection remains unclear. Although, studies have reported the antiviral and anti-inflammatory role of these drugs, however, clinical trial is still ongoing with early results showing no difference between these drugs and standard care [24–27]. The relationship between malaria and COVID-19 is complicated considering that malaria and COVID-19 have common symptoms, despite the fact that COVID-19 is more aggressive in adults while malaria affects more children [28].

There are several limitations to this study. Firstly, this is an ecological study and the outcome is limited with a high potential of bias. Secondly, the effect of lock downs, restriction of internal and international movement, restriction of public gatherings and public events, healthcare systems, public health surveillance and response to COVID-19 of each country and other population covariates were not adjusted in this study. Thirdly, the COVID-19 pandemic is still ongoing and the results may be different few months from now. Lastly, there is a possibility of underreporting of COVID-19 deaths in malaria endemic countries. COVID-19 testing in most malaria endemic regions such as Sub-Saharan Africa was not widespread and systematic. COVID-19 tests may not have been conducted for the majority of deaths that occurred in the community and in the health facility, hence, failure to identify deaths due to COVID-19 in these countries.

## Conclusion

In this study, a significant correlation was found between national malaria burden and COVID-19 mortality. Countries in Sub-Saharan Africa and Asia where malaria is prevalent reported lower COVID-19 deaths in contrast to the majority of European countries which have not recorded malaria infection with high number of COVID-19 deaths. Thus, exposure to malaria antigens and having anti-malarial immunity may be responsible for the reduced COVID-19 deaths in malaria endemic countries. Given the evidence from this ecological study, it may worth conducting laboratory experiments and clinical trials in order to validate the results of this study.

## Data Availability

The datasets used and/or analyzed during this study are available from the corresponding author on reasonable request.
